# Dendritic cells cross-presenting tumor-derived antigens are eliminated by invariant natural killer T (iNKT) cells, impairing development of anti-tumor immunity to a mouse carcinoma

**DOI:** 10.1186/2051-1426-1-S1-P117

**Published:** 2013-11-07

**Authors:** Karsten A Pilones, Joseph Aryankalayil, Silvia Formenti, Sandra Demaria

**Affiliations:** 1 Pathology, NYU School of Medicine, New York, NY, USA; 2 Radiation Oncology, NYU School of Medicine, New York, NY, USA

## 

iNKT cells are CD1d-restricted T cells that are rapidly activated in response to antigen and play a key role in promoting adaptive anti-tumor immunity. Surprisingly, we found that mice bearing 4T1 breast cancer CD8+ T cells inhibited spontaneous metastases in the absence of iNKT cells, suggesting that iNKT cells were suppressing the onset of CD8+ anti-tumor T cell responses. iNKT-deficient (iNKT-/-) mice also showed a markedly improved response to treatment with local radiotherapy (RT) and anti-CTLA-4 antibody (Pilones et al., Clin Cancer Res 2009). Moreover, administration of iNKT cell activator β-galactosyl-ceramide to wild type (WT) mice did not induce anti-tumor responses to 4T1. To understand the mechanisms of this unexpected iNKT immunosuppression, we analyzed dendritic cells (DC) in 4T1 tumor-bearing mice for differences that could explain the decreased priming of anti-tumor T cells in WT compared to iNKT-/- mice. Mice were inoculated subcutaneously with 4T1 tumor cells on day 0. Tumor and lymph nodes (LN) were analyzed for DC numbers and maturation. Mice were treated with RT+anti-CTLA-4 antibody, as previously described. To block CD1d in vivo, mice were given 3 doses of anti-CD1d mAb (Clone 20H2) on days 3, 7 and 11 post tumor inoculation. Mice were followed for tumor growth and survival, and long-term survivors were given a second tumor challenge to assess anti-tumor memory responses. In vitro cytotoxicity assays were used to determine if DC loaded with 4T1 tumor lysate were killed by iNKT cells. DC numbers were similar in healthy WT and iNKT-/- mice, while 4T1 tumor-bearing WT mice showed a significantly lower number of DC compared to iNKT-/- mice in the tumors (p=0.004) and draining LN (p<0.05) but not in non-draining LNs (p=0.782). Intratumoral DCs from iNKT-/- mice further showed increased expression of maturation markers compared to DC from WT mice. Blockade of CD1d restored DC numbers in tumor and draining LN of WT mice and markedly improved the therapeutic response to RT+anti-CTLA-4, with some mice showing complete tumor rejection and long-term protective memory responses. DCs loaded with 4T1 tumor lysates were killed by iNKT cells in vitro and killing was CD1d-dependent. 4T1 cells, which express minimal surface levels of CD1d, were resistant to NKT cell-mediated killing. Here we describe a novel mechanism of immune escape by tumors, namely the elimination by iNKT cells of DC cross-presenting tumor antigens in the tumor and draining LN. Data suggest that CD1d blockade may help overcome iNKT-mediated suppression and improve response of some tumors to immunotherapy.

